# Gleanings from the Journals, and Comments

**Published:** 1893-05

**Authors:** Edwin D. Downs

**Affiliations:** Owego, N. Y.


AMERICAN JOURNAL

—OF—

DENTAL SCIENCE.

VOL. XXVII. BALTIMORE, MAY, 1893. NO. I.

ARTICLE I.

### GLEANINGS FROM THE JOURNALS, AND COMMENTS.

BY EDWIN D. DOWNS, D. D. S., OWEGO, N. Y.

Read at the Twenty-fourth Annual Union Dental Convention of the Sixth, Seventh and Eighth District Dental Societies of the State of New York, held at Binghamton, October 24, 26 and 27, 1892.

The paper I am about to read was prepared for the last May meeting of our Sixth District Dental Society, and was written with an object which is set forth in the preamble. It was read at a session at which very few were present. Your Business Committee insisted upon my reading it here, although personally I did not think it was a proper paper to present to this Union Meeting. But at that time it looked as if they were to have very few papers, and it was held that this would introduce many subjects for discussion. Although I had already made some preparation towards a paper of a different character, I yielded to their request. If it seems a waste of valuable time, you must let them share the responsibility with me. The premable and the closing paragraphs I have allowed to stand just as then read, in explanation of my reasons for writing it. I mention this now, as these remarks were meant for that meeting, and were written after some hard work had been done in an effort to provide a programme.

The title of the paper is "Gleanings From the Journals, and Comments." Most of the "Gleanings" are as then written, but a few have been discarded, and one on methods of root filling added. With this explanation, I begin my paper here.

Before taking up the subject indicated by the title, I hope you will permit me to outline my reasons for writing such a paper. Having had, as you all know, some experience in arranging programmes for this society, I have found the chief difficulty in procuring papers is the idea which men have that they must write something elaborate, scientific and new. If the paper was to go before a body of scientific pretentions, a leader of dental thought, like our American Dental Association, for instance, whose proceedings are published and distributed to the whole dental world, then this might be true.

But we must remember that our societies are graded, and the ponderous thought of a society which gathers its members from the whole country, would be out of place in one whose membership is confined to a few counties, and composed largely of young men. The object of our meeting together annually is not simply to listen to papers, but to compare experiences, modestly stating our successes and frankly confessing our failures, getting in closer touch with our fellows, going home with renewed ambition to make better dentists of ourselves, and to awake us to the truth that all that is known of preserving teeth is not confined to the four walls of our own operating room, nor even to that of any single dentist, even though it be glittering with nickel-plate and improved machinery, or where beautifully engrossed and framed diplomas hang. While dentistry must make its advance to a liberal profession through the channels of original and scientific investigations, yet its daily work is full of laborious details, whose difficulties are solved by the ingenious rather than the scientific mind.

It often occurs to me, as I suppose it has to many of you when reading a dental journal, that I would like to talk its contents over with some one. Moreover, I often see a suggestion that I try, and find it in my hands a success or failure; when a failure, I do not always know whether the fault lies in me or the method, and I would like to hear of the experience of some one else, but do not always remember it when at our meetings.

Again, some things that I think I will try, in the busy life of our vocation slip from my mind before the opportunity occurs. But some one else may have tried it, and be able to approve or condemn it, for all ideas must stand the test of clinical experience to have their value proven. Therefore, as a part of our meeting, it has seemed to me that a paper of this character should be written every year by some member, and discussed by all, having virtually before us the literature of the year, and while the essayist may present but a small part of it, others will add to it, and possibly we will all read our journals better, and put their teachings more into daily practice for the sake of contributing our mite to the general information.

It is not my intention, and would not be if I had the whole year to prepare my paper, to give you a synopsis of even one dental journal, and so I shall only attempt to call your attention to a few of the things that have remained in my mind from the year's reading, confining myself mostly to practical subjects, otherwise I should occupy more than the whole time allowed for this meeting.

### DISINFECTION OF INSTRUMENTS.

While others have spoken words of value, Miller has covered the ground most effectually in an article in the *Cosmos*, for July, 1891, and also in his work on Micro-Organisms. If any one has failed to read his article in the *Cosmos*, he should do it at once, and those who have read it should re-read it, until its teachings are firmly impressed upon the mind. I find it hard work not to quote it entire, but a few sentences and a statement of his results must suffice here. He says, "It is true that there are still some whose appreciation of their duty toward those who commit themselves to their care is so stunted that they insist upon the right to spread infection by unclean instruments, or fingers that are not absolutely free from germs. Fortunately such men are rapidly becoming fewer, and will not be able to hold out long against the just condemnation of an advancing profession."

He adds later, "We cannot even touch any point in the oral cavity without our instruments becoming coated with a layer of infectious material."

For cleaning rubber dam, he says, the only safe way is boiling for fifteen minutes. Personally, it is a number of years since I washed and again used a piece of rubber dam.

I shall have to refer you to this article for details of his experiments, but his results certainly were surprising. Even concentrated carbolic acid failed to sterilize instruments, after an exposure ranging in some cases as high as twelve minutes. He did find that an exposure of from fifteen to twenty minutes in a five per cent. solution of bichloride of mercury could be depended upon in all but exceptional cases.

Letting alone the risk of injury to instruments, the time required, even with this solution, is a serious problem. But boiling all small instruments, such as are in use in our operating rooms, for three months, was found sufficient—forceps require five. The addition of soda prevents rusting.

If one will only make proper arrangements this becomes the easiest, as well as the quickest and most effective means of sterilization at our command.

### COPPER AMALGAM.

Last year Dr. Nelson gave us an interesting paper on copper amalgam. But I fear most of us have found its tendency to waste so great, that we have been obliged almost to abandon it, yet I still find that in teeth with a tendency to rapid recurrent decay, especially upon the buccal faces of molars, it seems to be of value. In such teeth, in such places, I have not found that it wastes materially, and I occasionally use it, and would not wish to leave it out of my case.

Another use I make of it is in regulating cases, when I desire to fit apparatus to teeth in a way that has a tendency to mar plaster. I make parts of the cast of this material. It will bear light swaging, can be employed in modeling compound impressions, and can be used over and over again, so that a small quantity kept for this purpose will suffice a man for a life time.

### COPPER, IN REGULATING CASES.

Dr. V. H. Jackson presents this in a valuable article in the December, 1891, *Cosmos*, where many simple and inexpensive apparatuses are illustrated. I have used copper in a few cases, and have found, as he says, that food does not decompose under it. I do not know whether the rest of you have noticed it, but I have, that occasionally teeth have bleached under regulating appliances. I do not believe that they do when copper is employed; and it is asserted that it has been used in connection with clasps on artificial dentures, and has seemed to prevent the destructive action of the clasp upon the teeth.

### MAT GOLD.

The opinions on this material vary as they do on nearly everything we use. A few cases have come to my knowledge in which it failed to give satisfaction, but in those which I have personally observed, it has appeared to me that too large pieces have been used. A dentist buys one single package of this, usually for trial, and quite likely the very first case he gets he tries it, regardless of whether the pieces are of an appropriate size or not. It should be used in pellets that can easily be carried to place, packing at first with rather broad-faced pluggers, with shallow serations, condensing with finer points. It is one of the rapid working golds, and I think all such must be used with care, but properly employed and in appropriate places, nothing in the form of gold has given me better satisfaction.

In the fall of 1897, I used considerable of it in heavy contour work. Many of the fillings then inserted have been constantly under observation. They seem hard, are free from pits, retain their bright polish, resist mastication, and up to the present time are satisfactory in every respect. I do not use this gold for everything, any more than I use gold itself for everything, but I do use it in a very large percentage of cases. It is very cohesive, yet soft and easily worked, and I think makes contour work possible, even to operators of moderate skill.

I really do not like that expression, for in my experience the contouring of a tooth is not nearly as difficult as the proper protection of the walls, but the quality of adhesiveness is necessary. I might add that I have used this gold with hand pressure and the automatic and electric mallets, giving great satisfaction with the latter, rather to my surprise.

### DEVITALIZING OF THE PULP.

As familiar as we all are with this subject, it occasionally presents difficulties. One of the valuable features of our last Union Meeting was the papers and discussions regarding it, all of which have since been published in the *Dental Practitioner* of Buffalo, and in the *Cosmos*. Dr. McCall's paper on the use of cocaine for this purpose induced me to test it in practice. After my first case I moistened the crystals with carbolic acid, and I have found that with care and a little time I have been able to remove pulps, causing no more pain than the preparation of a somewhat sensitive tooth for filling.

Possibly if I were a more courageous or skillful man, I might have learned to knock them on the head with a club (the pulps, not the patients), and so found less use for this method.

### METHODS OF ROOT-FILLING.

This is an old subject that crops up periodically, and while present methods seem satisfactory in most cases, yet it is one we shall probably continue to discuss, so long as we have pulpless teeth to treat.

I desire to call your attention to an article by Dr. Joseph Head, read before the Odontological Society of Pennsylvania, and published in the society's proceedings. I am unacquainted with any who are following the practice advocated, although it seemed to me then, and has since, that the method there taught was backed up with such tests as to make it worthy clinical trial and comparison. Any digest I can here make will not do the matter justice, but I will refer any one interested to his paper, and the discussion in the May, 1889, *International Dental Journal*, and the sequel to the discussion in the next number of the same journal. I will quote briefly his words to describe his method: "Put on the rubber dam, remove the dressings, and blow hot air into the tooth until it becomes painful. Then, using a hypodermic syring filled with warm carbolized cosmoline, pump the canals full. In dealing with large canals this will be an easy process. In those of a small diameter the passage of the cosmoline to the apex will be aided not only by capillary attraction, but also by the contraction of the cooling air. By finally pressing a pellet of cotton soaked in cosmoline over the small orifice, and then inserting a minute shred of cotton wherever possible, it seems reasonable to suppose that the canal can be filled to the apical foramen with an antiseptic sufficiently viscid to exclude moisture from without. Cotton should then be packed in the large canals to act as a support for the medicament.

"The canals should be filled with cotton to the pulp chamber, and a small pellet soaked in cosmoline placed over the orifices of those which are too small to allow the entrance of a thread. The cavity should now be washed with chloroform to remove superfluous grease, and the pulp chamber filled with gutta-percha or cement. I connect the mouths of the canals with protected cotton in order to expedite venting, should it be necessary. This is merely my personal preference. It is not essential. The filling to be used in covering the contents of the pulp chamber, of course, must vary according to the individual peculiarities of the tooth."

Dr. Head presented with his paper glass tubes filled with cement, gutta-percha, and by his method, which had been tested in aniline ink. All showed leakage except his own. His tubes were objected to, and it was suggested that he draw them to a fine point, file off the point, fill, and he would get different results. At the next meeting, he presented, not glass tubes, but natural teeth, filled under favorable conditions outside of the mouth. These had been tested as before, and the results were the same. I have never filled a root in the manner he advocates, although I have used to a limited extent, and with much satisfaction, a paste recommended by Dr. Von Woert, of Brooklyn, consisting of iodol, oxide of zinc and cosmoline. But this article has always been in my mind, and some things in my clinical experience have seemed to support the assertions of the essayist.

In removing root fillings of either cement or gutta-percha, I have noticed the odor, and although most of these teeth had given no trouble, and were, in some cases, crowned without any further medication than saturation at the moment with an antiseptic, and have continued to give no trouble, yet it has made me question the desirability of these materials for the purpose for which we use them, providing something better can be obtained.

I abandoned my practice rather suddenly once, for six months, on account of a surgical operation. At that time I had many teeth under treatment, which it was impossible for me to fill. These teeth were stopped with marine lint, dipped in a paste of iodoform and vaseline, the root having previously had some of the paste worked into it. The patients were advised to consult others, but many of them awaited my return, and although this was in the fall of 1887, some of those roots have only recently been opened to receive more permanent fillings, for these were only intended as dressings, and they have been filled without subsequent treatment or trouble.

The discussion of the paper seemed to turn on the use of cotton, and not sufficient allowance seemed to be made for the different conditions under which it was used. The roots having been first saturated or filled with carbolized cosmoline, the cotton saturated with the same offered an easy method of filling the large cavity. It was in fact a vehicle to carry and kept it there, and was besides a filling that could easily be removed in case of subsequent trouble. I know many will reply that if the work had been thoroughly done in the first place, retreat would not have been necessary, but whether a necessity or not; it is sometimes a prudent thing to arrange for.

What are the objections to cotton? Is not the principal one the danger of its absorbing moisture and permitting decomposition? Do gutta-percha and the cements absorb no moisture, and do they not allow decomposition? If cotton can be maintained in an aseptic, non-absorbent condition, would it not have advantages as a root filling? Do not understand me as advocating cotton, for I have never used it except as a vehicle, and that mostly in oxychloride fillings, and even if I were to fill roots as suggested I should probably use marine lint instead, believing it to possess some advantages over cotton. It is a lint saturated with tar, and used quite extensively in surgical dressings.

The question now naturally arises, does Dr. Head's method of using cotton prevent absorption of moisture, and thus maintain pulpless teeth in a healthy condition? The only personal experience I have to offer is to repeat what I said a few moments since, *i. e.*, marine lint with iodoform paste has kept pulpless teeth from giving trouble for years, when covered with gutta-percha, although not packed with any such intention, and the covering filling not inserted with the care that should have been given had they been expected to do the service they did.

Cosmoline is calculated, I believe, to resist and prevent moisture entering pulp canals. Cosmoline united with an antiseptic is calculated to prevent moisture entering a root, and to preserve it in aseptic condition if once made so. If this is true, have we not a use for such materials for root fillings?

Personally, I use gutta-percha almost exclusively for this purpose, and with such perfect satisfaction so far as subsequent trouble and ease in working is concerned, that I certainly shall continue to do so for the present, in the larger roots. But there is a class of roots so small and so difficult of access, that I do not feel sure that I have filled them, and here is where this method seems applicable. The ability to take a warm, heat-dissolved preparation and inject it into these roots, appeals to me strongly.

### ARGENTI NITRAS.

Probably no paper has appeared during the year that has shown more clearly the patience of the truly scientific mind than that by Dr. Stebbins, in the *International Dental Journal* for October, 1891, entitled, "Argenti Nitras as a Therapeutic Agent in Dentistry."

Here is a man who for six years carefully experimented and recorded his results, who did not rush into print with a half-supported theory, but finally, when time, the great factor in our operation had stamped its approval of his theory, appeared before the societies with his patients and his statistics. Whether one adopts the practice or not, we are bound to commend the spirit of the man. With wooden points and the mouth protected, he paints the cavities in certain cases without removal of decay, and in a large percentage no recurrence takes place. This treatment is particularly applicable to small, sensitive cavities on the buccal and labial faces of teeth, and to stop the progress of decay in the deciduous teeth. Of course its color is objectionable, and it cannot be used when the decay has reached the live pulp, as it will cause pain.

It might be well to mention that he rubs a little amalgam, or silver filings over the decay to take up the free nitric acid, and washes the mouth freely after the use of the remedy. My own experience with it is too limited to express an opinion founded on observation, but I do believe it is the proper treatment for the deciduous teeth, and will give our little patients and ourselves an amount of comfort that every dentist can appreciate, making the little ones love to come to us, when they may receive relief from suffering without the infliction of pain.

Nowhere, more than here, has the embarrassment that has extended throughout this paper assailed me. How many have read this paper? How many have not? How much do you wish me to tell you of this matter? Or is it so familiar to you that you wish me to stop? These are the questions that have tormented me from first to last. So I will leave the paper with you for discussion. Lengthy as it is, as many subjects as it touches upon, you will all recognize that many of the very good things that have been printed are not recorded here; also that I have touched but lightly upon most of the subjects, partly from want of time and partly for the reasons given a moment since. But you must remember that the paper has been written as an experiment; if it meets with your approval, if bringing before you thus second-handed a portion of the literature of the year for the purposes of discussion and comparison of experiences seems to you to add to the interests of our district meeting, then let us have some one repeat the effort each year, only with more careful preparation. If it does not, stamp it with your disapproval, for it has been written with the sole object of promoting discussion and thus adding to the interest of the meeting. As I write, I am fresh from an opinion of Dr. Barrett's, in an editorial in *The Dental Practitioner*, in which he truly says, that papers are not written for the sake of unlimited taffy, but for antagonism as well as approval.—*Dental Practitioner and Advertiser.*

